# As at home, so at work? The dynamic of relationship quality, work engagement, and burnout through the lens of basic psychological needs

**DOI:** 10.1371/journal.pone.0335404

**Published:** 2025-11-10

**Authors:** Lars van Tuin, Maria C.W. Peeters, Willem van Rhenen, Iris Arends, Jurriën den Hollander

**Affiliations:** 1 Arbo Unie, Knowledge Institute for Work and Health, Utrecht, The Netherlands; 2 Department of Social, Health and Organisational Psychology, Utrecht University, Utrecht, The Netherlands; 3 Human Performance Management Group, Department of Industrial Engineering and Innovation Sciences, Eindhoven University of Technology, Eindhoven, The Netherlands; 4 Center for Strategy, Organization and leadership Business Universiteit Nyenrode, Breukelen, The Netherlands; 5 Department of Health Sciences, Community & Occupational Medicine, University Medical Center Groningen, University of Groningen, Groningen, The Netherlands; Guangxi Normal University, CHINA

## Abstract

The present study examines the relationship between the quality of partner relationships and work engagement and burnout, considering the satisfaction and frustration of basic psychological needs within that private relationship. We argue that basic psychological needs mediate between the partner relationship and work, serving as an explanatory mechanism in that relationship. We hypothesized that when things go well at home and a person’s basic needs in the partner relationship are fulfilled, it positively associates with work engagement and negatively with burnout. In contrast, we expected that when basic psychological needs are frustrated, it would have a negative relationship with work engagement and a positive relationship with burnout. We analyzed data collected from 317 Dutch residents in committed partner relationships using structural equation modeling in a parallel mediation model. As hypothesized, the results indicate that a supportive partner relationship is associated with higher satisfaction of basic needs, which in turn enhances work engagement and decreases the risk of burnout. Contrary to our hypothesis, we found that the frustration of a partner’s basic psychological needs in the partner relationship was positively associated with increased energy for work (work engagement) and lower levels of exhaustion (burnout). Individuals may channel their energy into work when their partner relationship compromises their basic needs. Implications for practice and future research are discussed.

## Introduction

Research consistently shows that experiences at work and at home influence one another. Work engagement can foster positive emotions that spill over into partner relationships, while workaholism and burnout symptoms tend to reduce partner relationship satisfaction and contribute to stress in the home domain [1–3]. Conversely, employees may also carry positive feelings from their home lives into their work, resulting in higher levels of engagement at work [4]. These findings suggest that relationship quality and work-related states such as engagement and burnout are best understood as reciprocal and mutually reinforcing.

The COVID-19 pandemic has further highlighted this interdependence; remote and hybrid work arrangements blurred the boundaries between personal and professional life, making the role of partner relationships in employee well-being more salient [5,6]. Working from home for one, two, or more days a week has become a routine for many office employees and is sometimes even a benefit that employers highlight to attract or retain talent [7]. Due to this merging of work and personal life, the quality of one’s partner relationship may affect one’s well-being at work even more than before the pandemic [8].

Despite these developments, most studies document how work-related states spill over into the family domain; fewer have investigated the reverse: how the quality of partner relationships associates with work engagement and burnout. It is suggested that supportive and high-quality relationships may buffer against stress responses and promote engagement; however, systematic exploration of this dynamic remains scarce [9]. Understanding this relationship is increasingly important in light of the trend of working from home, as well as considering the rising levels of work-related psychosocial stress [10], globally declining engagement levels [11], and the growing prioritization of work-life balance over career advancement [12].

The present study examined the intricacies between personal relationships and professional well-being. Specifically, it examines the satisfaction of basic psychological needs in the partner relationship and how these needs mediate the relationship between relationship quality, work engagement, and burnout. Awareness of the interdependencies between relational and occupational well-being may support the development of more holistic approaches to fostering resilience, motivation, and sustainable performance in today’s evolving world of work.

### Self-determination theory and relationship quality

To explain how relationship quality influences work outcomes, the present study draws on Self-Determination Theory (SDT) [13]. The significance of SDT in partner relationships lies in the explanatory role of basic psychological need satisfaction in the experienced quality of interpersonal relationships [14]. SDT posits that all human beings share three universal basic psychological needs: autonomy, relatedness, and competence. The fulfillment of these needs is predictive of human well-being, regardless of individual differences, while the frustration of these needs is detrimental to well-being [13]. The need for autonomy involves a sense of volition, choice, and personal causation for the behaviors in which individuals engage [15]. Relatedness pertains to the need to feel cared for, care for others, feel at home in relationships with significant others, and belong [16]. The need for competence refers to feeling effective in one’s behaviors in playful exploration [17].

When the partner relationship is perceived as supportive of one’s sense of autonomy, it leads to fewer negative emotions and more positive behaviors [18]. Also, in the case of a relational conflict, it was found that autonomous reasons for being in the relationship and higher levels of need fulfillment of all three basic needs were predictive of satisfaction [19]. Moreover, a diary study revealed that autonomous reasons and need fulfillment in the relationship also contributed to the quality of the post-disagreement relationship [20]. The more one’s basic psychological needs are met, the easier it becomes to securely attach oneself and experience a close connection with one’s partner [21,22]. Overall, satisfying basic psychological needs is crucial for experiencing high-quality close relationships.

Opposite to the satisfaction of basic psychological needs, SDT posits the frustration of those needs. Need frustration is considered a separate concept from needs satisfaction and entails more than just not finding one’s needs for psychological need satisfaction met [23]. It involves a more active undermining of the partner’s needs, such as intentional need thwarting [24]. For instance, when partners feel pressured to behave in specific ways, the need for autonomy may be frustrated, which may concur with anger and irritation [21]. When a partner feels actively rejected, the need for relatedness is frustrated, which may coincide with emotions such as hurt and sadness [25]. In summary, it is expected that relationship quality positively relates to need satisfaction and negatively to need frustration. If relationship quality increases, it should reflect an increase in the satisfaction and a decrease in the frustration of one’s basic psychological needs. When the relationship quality drops, it should reflect a decline in satisfaction and an increase in the frustration of basic needs.

Studies in the domain of SDT also showed that the satisfaction and frustration of basic psychological needs often mediate the relationship between the quality of interpersonal relationships and health- and performance-related outcomes. Leadership studies found that an autonomy-supportive leadership style strengthens the employees’ sense of autonomy, competence, and relatedness, stimulating positive work outcomes and engagement [26] and that a leadership style characterized by controlling motives increased employees’ need frustration and was subsequently associated with poor work outcomes [27]. In sports coaching, athletes perform better and report higher psychological well-being when their coaches employ an autonomy-supportive coaching style that fulfills the athlete’s basic psychological needs [28]. When teachers in higher education adopt an autonomy-supportive approach that satisfies students’ basic needs, their motivation, well-being, and academic achievements benefit [29,30]. The same counts for parental autonomy support and children’s well-being and parental psychological control and children’s ill-being [31].

Through its mediating role, basic psychological needs theory adds explanatory value to the relationships under study [23,32]. SDT posits that basic psychological needs should be considered an underlying mechanism that explains the integration of extrinsic social elements and their impact on a person’s well-being and psychological growth [24,33]. Building on this theoretical and empirical work, we argue that if a partner relationship nurtures a person’s needs for autonomy, competence, and relatedness, it will facilitate positive integration and foster psychological well-being and growth, which may reflect on work-related states. If, in contrast, a private relationship frustrates or even thwarts a person’s basic psychological needs, it will lead to negative integration and ill-being.

### Relationship quality, work engagement, and burnout

In the present study, we conceptualize relationship quality as an individual’s perception of the extent to which their romantic partnership meets their emotional and practical needs, emphasizing mutual support, commitment, and positive relational behaviors [34]. This functional definition avoids conflating relationship quality with related but distinct constructs such as satisfaction, stability, or agreement on major life domains. Instead, it focuses on how the relationship contributes to overall psychosocial well-being. More practically, it represents an approach that emphasizes functionality in the relationship, rather than focusing on happiness or relationship problems, and allows for diverging preferences in committed partner relationships [35].

Work engagement, as developed by Schaufeli and colleagues, is defined as a positive, fulfilling work-related state characterized by vigor (high energy and mental resilience), dedication (a sense of significance, enthusiasm, and inspiration), and absorption (full concentration and immersion in one’s work) [36]. Yet engagement is not unconditionally beneficial. When taken to extremes, high levels of engagement can lead to work–family conflict and relational strain, particularly when employees become overly involved in their jobs [37]. Similarly, burnout—defined by exhaustion, cynicism, and reduced efficacy—not only impairs work functioning but also negatively affects partner relationships and family dynamics [38,39]. One longitudinal study found that reducing work-family conflict reduced burnout [40]. Another study showed that employees high on burnout score lower on positive affect and work engagement in reaction to negative external stressors [41]. Theoretically, high-quality romantic partner relationships may bolster work engagement by providing emotional stability, enhancing resilience, and reinforcing intrinsic motivation [9]. Simultaneously, they may act as protective factors against burnout by alleviating stress and reducing the accumulation of work-related strain.

By investigating how the satisfaction and frustration of basic needs within partner relationships may mediate the association between the quality of the partner relationship and work-related states, we contribute to a more nuanced understanding of how relationship quality can shape work-related outcomes.

### Hypotheses

H1. Relationship quality is positively associated with work engagement.

H2. Relationship quality is negatively associated with burnout.

H3. The satisfaction of basic psychological needs mediates the association between relationship quality and work outcomes, such that it (a) positively mediates the link with work engagement and (b) negatively mediates the link with burnout.

H4. The frustration of basic psychological needs mediates the association between relationship quality and work outcomes, such that it (a) negatively mediates the link with work engagement and (b) positively mediates the link with burnout.

**Fig 1:**
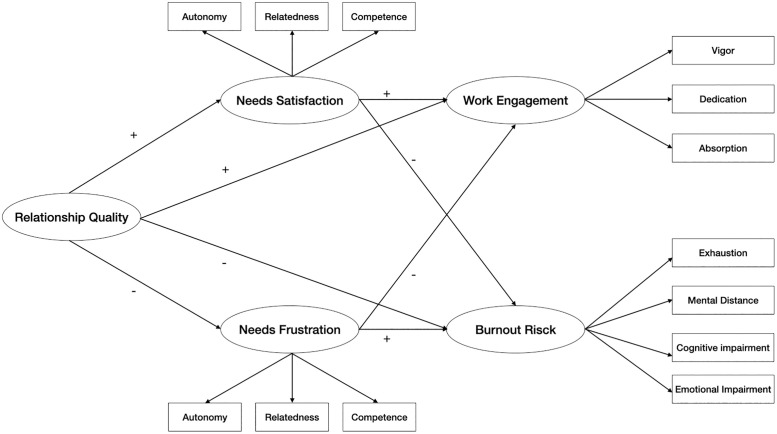
Study design.

We tested the hypotheses in a parallel mediation design, as depicted below in Fig 1.

## Materials and methods

### Sample

A self-report survey questionnaire was used to gather data from participants over 18 years old who were in committed relationships, worked at least eight hours per week, lived in the Netherlands, and were proficient in Dutch. Data were collected between September 20th, 2022, and December 20th, 2022. All participants provided written consent, confirming that they had understood the study’s information and voluntarily agreed to participate. Additionally, they consented to the study being used for scientific purposes and acknowledged that their information would be handled confidentially. Participants could stop completing the survey at any time and did not receive financial compensation. The online questionnaire comprised 89 items. We used a combination of convenience and snowball sampling. Convenience sampling was conducted via LinkedIn and Facebook, selecting participants who met the criteria, were available, and willing to participate. The additional snowball sampling helped us access participants through referrals in trusted social networks, which was important given the personal nature of the topic, resulting in N = 434 responses. After excluding incomplete responses, we retained N = 317 complete surveys.

The average age of the participants was 48 years (SD = 12), and 67% were female. On average, the respondents reported working 30 hours per week (SD = 10). Of the participants, 65% were in permanent employment, 67% were married, 7% had a registered partnership, 17% lived together without a registered partnership, and 9% reported not living together. Of all respondents, 95% reported being in monogamous relationships. The average duration of their relationships with their partner was 20 years (SD = 13). All participants had completed elementary education – the mandatory primary eight-year education in the Dutch schooling system between the ages of 4 and 12 – of which 10.5% did not pursue further studies, and 6% followed secondary vocational education. 38.5% had obtained higher vocational education, and 45% had pursued university studies.

Before the study, ethical clearance was obtained from the Ethical Review Board of Eindhoven University of Technology (ETC) granted on July 26, 2022, under reference number ERB2022IEIS21.

### Instruments

The following measures were applied. The *quality of partner relationships* was assessed using the 9-item survey developed and validated by Chonody et al. [34]. The instrument was selected for its strong psychometric properties, including high internal consistency and demonstrated convergent and known-groups validity. The scale was developed using a large, diverse community sample (N = 8,132) from the Enduring Love Project (a mixed methods study on long-term adult couple relationships), which spanned the United Kingdom, the United States, and Australia. The sample included a broad range of ages, sexual orientations, relationship types (e.g., married, cohabiting, dating), and ethnicities, making the scale especially suitable for diverse populations. It provides a strengths-based evaluation of relationship quality, emphasizing positive relationship aspects such as commitment, mutual enjoyment, and shared values, and employs inclusive language, making it appropriate for various relationship types. The Dutch version of the original English survey was constructed using Brislin’s [42] back-translation procedure. Items were rated on a 5-point Likert scale ranging from 1 (Completely untrue) to 5 (Completely true). An example item for the quality of a partner relationship is: ‘I am totally committed to making this relationship work.’ Reliability for the relationship quality scale was α = .91.

*Basic psychological need satisfaction and frustration* in romantic relationships were measured using the 24-item scale developed and validated by Vanhee et al. [43]. The scale assesses basic psychological need satisfaction and frustration through the subscales of autonomy, relatedness, and competence. An item for autonomy satisfaction is: ‘In my relationship with my partner, I feel that my decisions reflect what I really want.’ An item for autonomy frustration is: ‘In my relationship with my partner, most of the things I do feel like I have to do.’ Items were rated on a 5-point Likert scale ranging from 1 (completely untrue) to 5 (completely true). The reliability for satisfaction and frustration levels of autonomy was α = .85 and α = .87, respectively. An item for competence satisfaction is ‘I feel capable at what I do’ (α = .82). For competence frustration ‘I feel insecure about my capabilities’ (α = .89). An item for relatedness is ‘I feel connected with him/her’ (α = .93). For relatedness frustration ‘I feel that our relationship is just superficial’ (α = .88). The reliability for the satisfaction of basic psychological needs scale as a whole was α = .923 and α = .924 for needs frustration.

To measure *work engagement*, we used the shortened 9-item Utrecht Work Engagement Scale [44] with the subscales of vigor, dedication, and absorption (α = .94). The number of occurrences rated item questions over time: (0) Never (1) Once a year or less; (2) At least once a year; (3) At least once a month; (4) At least a couple of times a month; (5) At least once a week; (6) A couple of times per week or daily. An example item for vigor (α = .88) is: ‘At my job, I feel bursting with energy.’ An example item for dedication (α = .90) is: “I am proud of the work that I do.” For absorption (α = .85): “I get carried away when I am working.”

*Burnout* was measured with the shortened 12-item Burnout Assessment Tool (BAT) [45], measuring exhaustion, mental distance, cognitive impairment, and emotional impairment, showing an overall reliability score of (α = .87). Items were rated on a 5-point Likert scale ranging from 1 (never) to 5 (always). An example item for the exhaustion subscale (α = .81) is: “At work, I feel mentally exhausted.” An example item for mental distancing (α = .80) is “At work, I do not think much about what I am doing, and I function on autopilot.” An example of cognitive impairment (α = .79) is “At work, I struggle to think clearly.” An example of emotional impairment (α = .78) is “At work, I feel unable to control my emotions.”

Lastly, we added a one-item question about relationship problems on a 5-point scale from never to continuously: “Have relationship issues been affecting your personal situation last month?” (M = 1.40, SD = .80), because we suspected relationship issues may influence the results. Other variables we controlled for were age, average number of hours worked per week, and form of employment (permanent, temporary, or self-employed/entrepreneur).

## Results

### Preliminary analyses

We prepared the data for structural equation modeling (SEM) in JASP (Version 0.19.02). First, we estimated the correlation matrix (Table 1). The correlation matrix shows that the quality of the partner relationship strongly correlates with the satisfaction and frustration of basic psychological needs. The correlations between quality of the partner relationship and the work engagement subscales only indicated a significant correlation with vigor (**p* *< .05). The correlation between quality of the partner relationship and burnout is more robust, showing two significant correlations for exhaustion (**p* *< .01) and cognitive impairment (*p < .05*). The other correlations from relation quality to the subscales of work engagement and burnout were nonsignificant.

**Table 1:** Pearson’s correlation matrix.

Variable	M	SD	RQ	NDS	NDF	VIG	DED	ABS	EXH	MDI	CIP	EIP	PROB	AGE	WRKWK	EMPL
Relation Quality (RQ)	4.18	0.65	(0.91)													
Needs Satisfaction (NDS)	4.23	0.58	0.87***	(0.92)												
Needs Frustration (NDF)	1.74	0.66	−0.80***	−0.85***	(0.92)											
Vigor (VIG)	5.34	1.24	0.12*	0.23***	−0.24***	(0.88)										
Dedication (DED)	5.61	1.29	0.06	0.19**	−0.17**	0.81***	(0.90)									
Absorption (ABS)	4.87	1.39	0.07	0.13*	−0.12*	0.74***	0.77***	(0.85)								
Exhaustion (EXH)	2.27	0.82	−0.16**	−0.25***	0.28***	−0.61***	−0.45***	−0.35***	(0.81)							
Mental Distance (MDI)	1.81	0.78	−0.06	−0.17**	0.24***	−0.70***	−0.71***	−0.54***	0.55***	(0.80)						
Cognitive Impairment (CIP)	2.11	0.66	−0.14*	−0.21***	0.19***	−0.41***	−0.38***	−0.39***	0.37***	0.45***	(0.79)					
Emotional Impairment (EIP)	1.70	0.62	−0.11	−0.23***	0.26***	−0.32***	−0.35***	−0.22***	0.33***	0.50***	0.36***	(0.78)				
Relationship Problems (PROB)	1.40	0.80	−0.58***	−0.54***	0.65***	−0.04	0.00	−0.01	0.05	0.07	0.11	0.09	—			
Age (AGE)	47.56	12.05	−0.08	−0.05	−0.02	0.23***	0.16**	0.09	−0.22***	−0.23***	−0.16**	−0.00	−0.06	—		
Hrs. Worked/week (WRKWK)	30.46	10.30	0.08	0.09	−0.09	0.17**	0.11	0.17**	0.01	−0.05	0.00	−0.03	0.03	−0.05	—	
Employment (EMPL)	1.67	1.02	0.05	0.07	−0.05	0.17**	0.21***	0.17**	−0.11*	−0.09	−0.08	−0.12*	−0.00	0.11	0.12*	—

Significance * p < .05, ** p < .01, *** p < .001. (WRKWK) is the average number of hours worked per week, (EMPL) is permanent- temporary employment or self-employed/entrepreneur M = Mean, SD = Standard Deviation. On the diagonal, Cronbach’s alpha is depicted.

### Measurement model

We then tested how the measurement model fitted the data by conducting a confirmatory factor analysis in the structural model in JASP and entered the syntax in lavaan. Our initial model (Fig 1) measured all scales as one factor: Relationship Quality (RQ), Needs Satisfaction (NDS), Needs Frustration (NDF), Work Engagement (WE), and Burnout (BO). We set the model estimation to WLSMV (weighted least square mean and variance-adjusted), which is a robust estimation method that does not require a normal distribution of data [46] and operates on the assumption that continuous latent responses follow a multivariate normal distribution [47]. Additionally, we selected the option to obtain additional fit measures. Model 1 fitted the data to an acceptable level, considering the general rules of thumb for assessing model fit. The chi-squared divided by the degrees of freedom was χ^2^/**df* *< 3, but the CFI (Comparative Fit Index,.89) and TLI (Tucker-Lewis Index,.88) were < .90 [48]. Also, the RMSEA (Root Mean Square Error of Approximation (.064) was > .06. And, although the width of the confidence interval was small (.060 −.068) it returned significant where non-significance is preferred when estimating PCLOSE, the *p*-value of close fit [49,50]. Hence, we continued testing a second model for a better model fit. We estimated the dependent variables for work engagement and burnout through their subscales of vigor, dedication, and absorption for work engagement. For burnout, these were the subscales of exhaustion, mental distancing, and cognitive and emotional impairment. Model 2 had 10 factors against five for model 1 and showed a good fit to the data: CFI (.92) and TLI (.91) were > .90, the RMSEA showed a closer fit (.051), and the PCLOSE returned nonsignificant (**p* *= .285).

### Hypothesis testing

**Fig 2:**
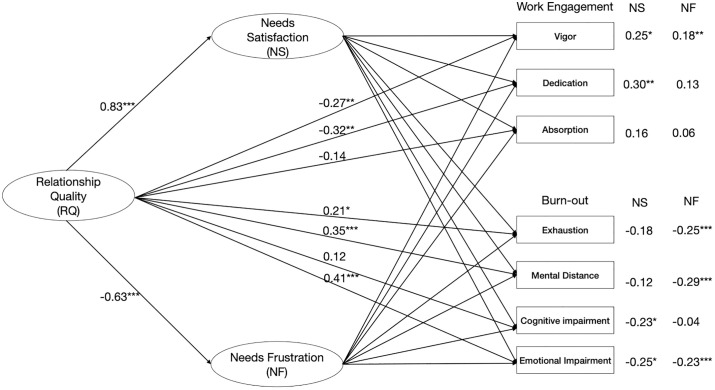
Model 2, direct and indirect effects.

To test the hypotheses, we estimated model 2 in a parallel mediation model in JASP SEM with standardized estimates, a 95% confidence interval, and 5.000 bootstrap replications set to the bias-corrected percentile. Additional parameters were the total indirect effects and the standardized path coefficients (please see the supporting information S1 Table for all path coefficients). First, we examined the direct effects of the quality of the partner relationship on the satisfaction and frustration of basic psychological needs and on work engagement and burnout (Fig 2). We found a significant and positive path from the quality of the partner relationship to the satisfaction of basic needs and a negative path to the frustration of these needs. In contrast, and contrary to our assumptions about the positive path from quality of the partner relationship to work engagement, we found a negative path to vigor and dedication, and no association with absorption. We had to reject H1. The path from the quality of the partner relationship to burnout was positive and significant for exhaustion, mental distance, and emotional impairment, leading to rejecting H2 as well, as we hypothesized a negative path.

We then studied the indirect effects via the satisfaction of basic psychological needs and the frustration of those needs to test hypotheses 3 and 4. The results showed that the signs of the direct and indirect paths for the quality of the partner relationship to work engagement and burnout via need satisfaction were opposite. Usually, a mediation model’s direct and indirect effects share the same sign. There may be a suppression effect in the case of opposite signs [51]. We followed the procedure described by MacKinnon et al. [52] to establish whether there was a suppression effect in this mediation. In short, the procedure consists of finding whether the population’s third variable effect is zero, positive, or negative (⍺β = τ – τ’) and then calculating the difference between the total effect τ and the direct effect τ’ (Table 2). The analysis indicated that our model was a mediation model, and there was no suppression. Next, we conducted the Sobel test for the standard errors and calculated the confidence intervals (Table 3). We found that need satisfaction mediates the relationship between the quality of the partner relationship and work engagement for vigor and dedication (H3a) and with burnout for cognitive and emotional impairment (H3b). This confirmed the indirect effects we found in the mediation analysis (Fig 2). For need frustration, we found confirmation of the mediation of the association between the quality of the partner relationship and work engagement (H4a, vigor) and burnout (H4b, exhaustion, mental distance, and emotional impairment). However, contrary to our hypothesis, we found that need frustration associated positively with vigor and negatively with three of the four levels of burnout. Hence, we had to reject both hypotheses H4a and b.

**Table 2:** Mediation or suppression for needs satisfaction & frustration.

	τ	τ‘		(τ – τ‘)	⍺β (Satis.)	⍺β (Frust.)
Vigor	0.16	−0.27	τ > τ‘	0.43	0.245	0.183
Dedication	0.10	−0.32	τ > τ‘	0.43	0.301	0.125
Absorption	0.08	−0.14	τ > τ‘	0.23	0.164	0.064
Exhaustion	−0.22	0.21	τ < τ‘	−0.44	−0.183	−0.253
Mental distance	−0.06	0.35	τ < τ‘	−0.41	−0.125	−0.287
Cognitive impairment	−0.15	0.12	τ < τ‘	−0.27	−0.232	−0.039
Emotional impairment	−0.08	0.41	τ < τ‘	−0.48	−0.250	−0.234

τ = total effect;*.* τ’ = direct effect; ⍺β(Satis.) = indirect path coefficient for need satisfaction; ⍺β(Frust.) = indirect path coefficient for need frustration.

**Table 3:** Confidence interval estimation of the mediation effect using the Sobel test.

	*Need Satisfaction*				*Need Frustration*			
			95% Conf. Int.				95% Conf. Int.	
	Sobel	αβ (Satis.)	Lower	Upper	Sobel	αβ (Frust.)	Lower	Upper
Vigor	0.101	0.245	0.047	0.442	0.068	0.183	0.049	0.316
Dedication	0.103	0.301	0.099	0.504	0.070	0.125	−0.012	0.261
Absorption	0.108	0.164	−0.048	0.375	0.073	0.064	−0.078	0.206
Exhaustion	0.101	−0.183	−0.382	0.015	0.069	−0.253	−0.388	−0.118
Mental distance	0.104	−0.125	−0.328	0.079	0.070	−0.287	−0.424	−0.150
Cognitive impairment	0.108	−0.232	−0.444	−0.020	0.073	−0.039	−0.182	0.104
Emotional impairment	0.106	−0.250	−0.457	−0.043	0.071	−0.234	−0.374	−0.095

Sobel test applied was σ^2^_αβ _= σ^2^_α_β^2 +^ σ^2^_β_α^2^; αβ(Satis.) = indirect path coefficient for need satisfaction; αβ(Frust.) = indirect path coefficient for need frustration.

## Discussion

The present study examined the association between the quality of partner relationships, work engagement, and burnout. It specifically focused on the mediating role of the satisfaction and frustration of basic psychological needs within these partner relationships. Data analysis revealed that relationship quality was negatively associated with work engagement and positively associated with burnout, which did not support hypotheses H1 and H2. This outcome was primarily influenced by the mediating effects of psychological need satisfaction and frustration, which reversed the expected direction of the relationships. We found support for hypothesis H3, which suggests that when a partner relationship meets an individual’s basic needs for autonomy, competence, and relatedness, the person is likely to feel more energized at work and less prone to exhaustion. Additionally, and contrary to hypothesis H4, need frustration did not negatively impact work outcomes. Instead, it was related to increased vigor and decreased burnout.

These results confirm that high-quality partner relationships that fulfill the partner’s basic psychological needs provide necessary psychological resources that extend into the work domain. These findings complement previous research, which has demonstrated that positive work experiences can foster engagement at home and that negative work-related experiences may lead to work-family conflict [1–4]. Employees who experience need satisfaction in their intimate partner relationships seem better equipped to bring energy, focus, and motivation into their professional roles, while being less prone to the symptoms of burnout.

In contrast to what we expected and hypothesized, the frustration of basic psychological needs was not linked to poorer outcomes but instead predicted higher vigor and lower burnout. The literature offers two potential explanations for this unexpected outcome. First, individuals may pursue extrinsic goals as a substitute for the frustration of basic psychological needs within their partner relationship. Within SDT, this phenomenon is referred to as need substitutes [53,54]. Second, they may seek alternative fulfillment of their intrinsic needs in another life domain, such as work. We will discuss these two approaches of extrinsic need substitutes and intrinsic orientations below.

Prior research has demonstrated that frustration of basic psychological needs within a partner relationship is associated with lower perceived relationship quality, increased conflict frequency, and altered communication patterns during disputes [55]. Specifically, the frustration of relational needs—particularly autonomy and relatedness—elicits negative emotions such as irritation, anger, sadness, and hurt [25], which can undermine a person’s self-worth and contribute to feelings of insecurity. According to self-determination theory (SDT), individuals experiencing such need frustration may attempt to compensate by seeking external sources of validation, including social status, popularity, material success, or career achievements [56,57]. This compensatory pursuit of need substitutes mitigates self-esteem threats and may support the individual in restoring a sense of self-worth [20]. SDT further posits that pursuing extrinsic goals as a substitute for need thwarting or frustration represents a maladaptive coping mechanism. Accordingly, individuals who perceive their partner relationship as lower in quality due to the frustration of their basic psychological needs may be more inclined to immerse themselves in their work as a need substitute, potentially explaining the observed increase in vigor and decrease in exhaustion in the present study. The literature also suggests that the benefits of pursuing extrinsic goals under internal pressures do not outweigh the long-term costs [58]. While working harder for extrinsic reasons may provide temporary satisfaction upon goal attainment, it often leads to a self-perpetuating cycle in which individuals must exert increasing effort to sustain that satisfaction [59]. Consequently, the observed increase in vigor when relational needs are frustrated at home may induce forms of work engagement characterized by excessive and compulsive work behaviors, as described in the workaholism literature [37].

It is also possible that, unlike the home environment, the energy for work increases because the workplace satisfies an individual’s basic psychological needs. People may find that their work serves as a positive outlet for their motivational drives, leading them to discover meaning and significance in their roles at work [60]. In these instances, the workplace provides a positive source of energy, bolstering employee well-being, enhancing work engagement, and decreasing burnout [61]. As a consequence, there is no need for the individual to seek extrinsic substitutes for unmet or frustrated needs because the person can find alternative and positive ways to fulfill their basic psychological needs at work. Like personal relationships, work is a consummate place for people to find meaning, satisfy the basic psychological needs for autonomy, relatedness, and competence, and realize intrinsic goals such as personal growth and a sense of contribution [57].

The present study contributes to our understanding of how basic psychological needs theory relates to the interplay between romantic relationships and work-related experiences. The post-COVID-19 blurring of boundaries between work and personal life makes it more likely that the quality of one’s relationship with one’s partner intersects with one’s energy and commitment to work, as well as the risk of experiencing burnout. Increasing levels of sick leave due to psychosocial work-related stress in recent years may highlight the importance of research in this area [62,63].

### Strengths, limitations, and further research

The present study has notable strengths. As far as we were able to ascertain, this is the first study to explore the relationship between partner relationship quality and work engagement through the lens of basic psychological needs theory. The study’s design and specifically the examination of the mediating role of both basic needs satisfaction and needs frustration helps to gain insights into the dynamics between the quality of the partner relationship and work outcomes. Despite these strengths, several limitations should be considered. First, the study’s cross-sectional design precludes any conclusions about causality. While the findings suggest a relationship between quality of the partner relationship and work engagement through basic needs frustration and satisfaction, the directionality of these effects remains uncertain. Future longitudinal and experimental studies are needed to clarify whether relational need frustration leads to increased work investment or, for example, whether pre-existing tendencies toward high work engagement influence perceptions of relationship quality. Also, the role of need satisfaction in the partner relationship and its relation with work engagement and burnout would benefit from a longitudinal design. It would be of interest to know how this association may fluctuate over longer time periods.

Second, we relied on self-report measures, which can introduce the risk of common method bias. This bias might inflate or distort the relationships we observed [64]. To mitigate this potential issue ex ante, we utilized well-researched and validated psychometric instruments with clear and neutrally worded items. Ex post, we conducted Harman’s single-factor test, which revealed that the first factor accounted for 39% of the total variance, a figure well below the commonly accepted threshold of 50% [65]. We also performed a common latent factor analysis (CLF) using confirmatory factor analysis (CFA), comparing the applied measurement model with a modified model that incorporated a common latent factor allowed to load on all observed variables. The inclusion of the CLF did not significantly improve model fit (ΔCFI = .012, ΔRMSEA = .002), and the variance extracted by the common latent factor was AVE = 0, indicating that common method variance is unlikely to affect the validity of our findings [64].

Third, the generalizability of the findings is limited. Although we controlled for background variables our sample characteristics highlight potential constraints on generalizability: 45% of participants held a university degree, with an average age of 48 years (SD = 12) and an average relationship length of 20 years (SD = 13). This raises the question of whether the same patterns would emerge in younger generational cohorts, particularly individuals under 35 in relationships shorter than five or six years. Furthermore, the study sample was limited to Dutch-speaking citizens, which may restrict the applicability of our findings to populations with different cultural backgrounds or work environments. Variations in relationship norms, occupational structures, occupational categories, and socio-cultural influences on work engagement may yield different outcomes in other research contexts. Future research should prioritize replication in diverse samples and cross-cultural studies to assess the robustness, ecological validity, and generalizability of these effects. A last aspect that may limit the generalizability of our findings was the use of a convenience sample and snowball sampling, which brings the risk of selection bias, as participants often refer to similar others, which may lead to sample homogeneity.

Ultimately, the study confirms the prevailing notion that a strong partner relationship fosters work engagement and mitigates burnout. However, it challenges the assumption that a bad partner relationship that thwarts one’s basic needs decreases engagement and increases burnout. Nonetheless, the present study does not account for individual differences in coping strategies. Some individuals may seek refuge in work to compensate for relational distress. In contrast, others may disengage from work and personal commitments altogether. There are myriad ways of dealing with adverse circumstances in the relationship with one’s partner at home. In sum, this study underscores the complexity of the relationship between partner relationship quality and work engagement through the lens of basic psychological needs theory. Addressing the outlined limitations through longitudinal research, diary studies, and multi-method approaches will be essential to disentangle the mechanisms at play further.

### Practical implications

The recent rise in work-related stress, increased employee absenteeism due to psychosocial factors, the growing number of burnouts, and the decline in work engagement make it essential for employers to safeguard the psychological well-being of their employees [11,66]. A recent survey of 26.000 employees across 35 countries showed that workers value a healthy work-life balance over pay or career advancement [12]. Also, employee retention rates and turnover intentions were found to correlate with work-life balance and the measures organizations take to support their staff. A recent study published in Nature revealed that hybrid work arrangements increased employee retention without negatively affecting performance and opportunities for promotion [67].

However, the blurring of boundaries between work and home—especially since the pandemic—has made employee well-being more complex and interdependent across life domains. While promoting work-life balance through flexible arrangements and the right to disconnect is essential [68], organizations should recognize that these measures alone may not be sufficient. Training managers to develop their capacity to recognize early warning signals—such as exhaustion, disengagement, or sudden shifts in motivation—and to engage in respectful, autonomy-supportive conversations with employees may be critical skills that help address issues before they escalate.

Expanding the role of managers in supporting employee well-being inevitably raises questions about the ethical boundaries of such involvement. Some may argue that such involvement intrudes on privacy and goes beyond the legitimate scope of managerial responsibility. Others may contend that when signs of distress—such as exhaustion, disengagement, unexpected spikes in energy, or a sudden aspiration for career advancement—become apparent at work, a sensitive and autonomy-supportive conversation that gently explores underlying factors, including relational well-being is both ethical and necessary [69].

## Conclusion

The present study examined the mediating role of the satisfaction and frustration of basic psychological needs in the association between the quality of partner relationships and work engagement and burnout. As expected, when one’s basic psychological needs in the relationship are satisfied, it was positively associated with work engagement and negatively with burnout. Contrary to our initial assumptions, the results showed a positive relationship between need frustration and work engagement (vigor), along with a negative correlation to burnout. We identified two potential explanations for this outcome within self-determination theory. First, when an individual’s basic psychological needs are frustrated in their partner relationship, they may seek extrinsic compensation through their work. This compensation can take the form of career advancement, social status, and financial progress as a substitute for those unfulfilled intrinsic needs. Second, in a more positive sense, work could provide an environment where individuals feel valued and appreciated, allowing their basic psychological needs to be met, in stark contrast to their experiences at home.

The blending of personal and professional lives in the wake of the pandemic necessitates that the organization’s leaders recognize the potential impact of employees’ personal relationships on their well-being and work engagement. Promoting well-being requires more than improving job design or offering flexible work arrangements. Managers should develop the skills to recognize early signs of strain, learn to engage in autonomy-supportive conversations, or even learn when to refer to professional resources while respecting the employees’ privacy and autonomy.

The study’s results prompt a deeper exploration of the complex, bidirectional relationships among the quality of the partner relationship, work engagement, and burnout. Future longitudinal and multi-method research is necessary to clarify the long-term effects of compensatory work engagement in response to relational need frustration, as well as to examine variations across different occupational and cultural contexts.

## Supporting information

S1 Table Path coefficients. (XLSX)

## References

[1] Schaufeli WB, Taris TW, Van Rhenen W. Workaholism, Burnout, and Work Engagement: Three of a Kind or Three Different Kinds of Employee Well‐being?. Applied Psychology. 2008;57(2):173–203. doi: 10.1111/j.1464-0597.2007.00285.x

[2] Bakker AB, Demerouti E, Burke R. Workaholism and relationship quality: a spillover-crossover perspective. J Occup Health Psychol. 2009;14(1):23–33. doi: 10.1037/a0013290 19210044

[3] Bakker AB, Demerouti E, Schaufeli WB. The crossover of burnout and work engagement among working couples. Human Relations. 2005;58(5):661–89. doi: 10.1177/0018726705055967

[4] Montgomery AJ, Peeters MCW, Schaufeli WB, Den Ouden M. Work-home interference among newspaper managers: its relationship with burnout and engagement. Anxiety, Stress & Coping. 2003;16(2):195–211. doi: 10.1080/1061580021000030535

[5] Chan XW, Kinman G. Work and non-work boundary management including remote and hybrid working. Wellbeing at Work in a Turbulent Era. Edward Elgar Publishing. 2024. 56–75. doi: 10.4337/9781035300549.00008

[6] Moreira A, Encarnação T, Viseu J, Au-Yong-Oliveira M. Conflict (Work-Family and Family-Work) and Task Performance: The Role of Well-Being in This Relationship. Administrative Sciences. 2023;13(4):94. doi: 10.3390/admsci13040094

[7] Ardi A, Cahyadi H, Meilani YFCP, Pramono R. Talent attraction through flexible work anytime from anywhere. J Infras Policy Dev. 2024;8(3). doi: 10.24294/jipd.v8i3.2998

[8] Chan XW, Shang S, Brough P, Wilkinson A, Lu C. Work, life and COVID‐19: a rapid review and practical recommendations for the post‐pandemic workplace. Asia Pac J Human Res. 2023;61(2):257–76. doi: 10.1111/1744-7941.12355

[9] Peplińska A, Godlewska-Werner D. Work engagement, emotional attitude to work and quality of relations in “early” and “late” dual career couples: the mediating role of satisfaction with one’s achievements. Health Psychol Rep. 2023;11(2):134–44. doi: 10.5114/hpr/165916 38084313 PMC10670788

[10] Palmucci DN, Santoro G. Managing employees’ needs and well-being in the post-COVID-19 era. Manag Decis. 2024;62: 4138–47. doi: 10.1108/md-02-2024-0233

[11] Gallup. State of the global workplace. Gallup. 2025. https://www.gallup.com/workplace/349484/state-of-the-global-workplace.aspx

[12] Randstad. Work-life balance tops pay: Randstad’s workmonitor reveals new workplace baseline. Workmonitor. 2025. https://www.randstad.com/press/2025/work-life-balance-tops-pay-randstads-workmonitor-reveals/

[13] Ryan RM, Deci EL. Self-determination theory. Guilford Publications. 2017.

[14] Vansteenkiste M, Ryan RM, Deci EL. Self-Determination Theory and the Explanatory Role of Psychological Needs in Human Well-Being*. Capabilities and Happiness. Oxford University PressOxford. 2008. 187–223. doi: 10.1093/oso/9780199532148.003.0009

[15] Charms R d e. Personal causation. Academic Press. 1968.

[16] Baumeister RF, Leary MR. The need to belong: Desire for interpersonal attachments as a fundamental human motivation. Psychological Bulletin. 1995;117(3):497–529. doi: 10.1037/0033-2909.117.3.497 7777651

[17] White RW. Motivation reconsidered: the concept of competence. Psychol Rev. 1959;66:297–333. doi: 10.1037/h0040934 13844397

[18] Knee CR, Patrick H, Vietor NA, Nanayakkara A, Neighbors C. Self-Determination as Growth Motivation in Romantic Relationships. Pers Soc Psychol Bull. 2002;28(5):609–19. doi: 10.1177/0146167202288005

[19] Knee CR, Lonsbary C, Canevello A, Patrick H. Self-determination and conflict in romantic relationships. J Pers Soc Psychol. 2005;89(6):997–1009. doi: 10.1037/0022-3514.89.6.997 16393030

[20] Patrick H, Knee CR, Canevello A, Lonsbary C. The role of need fulfillment in relationship functioning and well-being: a self-determination theory perspective. J Pers Soc Psychol. 2007;92(3):434–57. doi: 10.1037/0022-3514.92.3.434 17352602

[21] La Guardia JG, Patrick H. Self-determination theory as a fundamental theory of close relationships. Canadian Psychology / Psychologie canadienne. 2008;49(3):201–9. doi: 10.1037/a0012760

[22] Knee CR, Porter B, Rodriguez LM. Self-Determination and Regulation of Conflict in Romantic Relationships. Human Motivation and Interpersonal Relationships. Springer Netherlands. 2014. 139–58. doi: 10.1007/978-94-017-8542-6_7

[23] Chen B, Vansteenkiste M, Beyers W, Boone L, Deci EL, Van der Kaap-Deeder J, et al. Basic psychological need satisfaction, need frustration, and need strength across four cultures. Motiv Emot. 2014;39(2):216–36. doi: 10.1007/s11031-014-9450-1

[24] Vansteenkiste M, Ryan RM. On psychological growth and vulnerability: Basic psychological need satisfaction and need frustration as a unifying principle. Journal of Psychotherapy Integration. 2013;23(3):263–80. doi: 10.1037/a0032359

[25] Pirrone D, Sels L, Verhofstadt L. Relational needs frustration: an observational study on the role of negative (dis)engaging emotions. Front Psychol. 2023;14:1232125. doi: 10.3389/fpsyg.2023.1232125 38078212 PMC10701550

[26] Van den Broeck A, Slemp GR. Leadership. The Oxford Handbook of Self-Determination Theory. Oxford University Press. 2023. 920–38. doi: 10.1093/oxfordhb/9780197600047.013.47

[27] van Tuin L, Schaufeli WB, van Rhenen W. The Satisfaction and Frustration of Basic Psychological Needs in Engaging Leadership. J of Leadership Studies. 2020;14(2):6–23. doi: 10.1002/jls.21695

[28] Mossman LH, Slemp GR, Lewis KJ, Colla RH, O’Halloran P. Autonomy support in sport and exercise settings: a systematic review and meta-analysis. International Review of Sport and Exercise Psychology. 2024;17(1):540–63. doi: 10.1080/1750984x.2022.2031252

[29] Vite A, Patall EA, Chen M. Relationships Between Experiences of Autonomy and Well(Ill)-Being for K-12 Youth: A Meta-Analysis. Educ Psychol Rev. 2024;36(4). doi: 10.1007/s10648-024-09967-x

[30] Hutomo BA, Kurniawati F. Importance of Basic Psychological Needs Satisfaction in Higher Education: A Systematic Literature Review. G-Couns. 2024;9(1):233–46. doi: 10.31316/gcouns.v9i1.6326

[31] Bradshaw EL, Duineveld JJ, Conigrave JH, Steward BA, Ferber KA, Joussemet M, et al. Disentangling autonomy-supportive and psychologically controlling parenting: A meta-analysis of self-determination theory’s dual process model across cultures. Am Psychol. 2025;80(6):879–95. doi: 10.1037/amp0001389 39052356

[32] Ryan RM, Deci EL, Vansteenkiste M. Self-determination theory and the explanatory role of psychological needs in human well-being. 2006.

[33] Niemiec CP, Lynch MF, Vansteenkiste M, Bernstein J, Deci EL, Ryan RM. The antecedents and consequences of autonomous self-regulation for college: a self-determination theory perspective on socialization. J Adolesc. 2006;29(5):761–75. doi: 10.1016/j.adolescence.2005.11.009 16412502

[34] Chonody JM, Gabb J, Killian M, Dunk-West P. Measuring Relationship Quality in an International Study: Exploratory and Confirmatory Factor Validity. Res Soc Work Pract. 2018;28(8):920–30. doi: 10.1177/1049731516631120 30369776 PMC6187488

[35] Hull KE, Meier A, Ortyl T. The Changing Landscape of Love and Marriage. Contexts (Berkeley Calif). 2010;9(2):32–7. doi: 10.1525/ctx.2010.9.2.32 25435828 PMC4244648

[36] Schaufeli WB, Salanova M, González-romá V, Bakker AB. The Measurement of Engagement and Burnout: A Two Sample Confirmatory Factor Analytic Approach. Journal of Happiness Studies. 2002;3(1):71–92. doi: 10.1023/a:1015630930326

[37] Taris TW, de Jonge J. Workaholism: Taking Stock and Looking Forward. Annu Rev Organ Psychol Organ Behav. 2024;11(1):113–38. doi: 10.1146/annurev-orgpsych-111821-035514

[38] Russo E, Atroszko P, Zaniboni S, Toderi S, Balducci C. The Relationship between Workaholism and Personal Burnout in Dual-Earner Couples: An Analysis Using the Actor-Partner Interdependence Model. Sustainability. 2023;15(17):13009. doi: 10.3390/su151713009

[39] Bakker AB, Petrou P, Tsaousis I. Inequity in work and intimate relationships: a Spillover-Crossover model. Anxiety Stress Coping. 2012;25(5):491–506. doi: 10.1080/10615806.2011.619259 22059998

[40] Otto MCB, Van Ruysseveldt J, Hoefsmit N, Dam KV. The Development of a Proactive Burnout Prevention Inventory: How Employees Can Contribute to Reduce Burnout Risks. Int J Environ Res Public Health. 2020;17(5):1711. doi: 10.3390/ijerph17051711 32151047 PMC7084396

[41] Fleuren BPI, Nübold A, Uitdewilligen S, Verduyn P, Hülsheger UR. Troubles on troubled minds: an intensive longitudinal diary study on the role of burnout in the resilience process following acute stressor exposure. European Journal of Work and Organizational Psychology. 2023;32(3):373–88. doi: 10.1080/1359432x.2022.2161369

[42] Brislin RW. Back-Translation for Cross-Cultural Research. Journal of Cross-Cultural Psychology. 1970;1(3):185–216. doi: 10.1177/135910457000100301

[43] Vanhee G, Lemmens GMD, Verhofstadt LL. Relationship satisfaction: High need satisfaction or low need frustration?. soc behav pers. 2016;44(6):923–30. doi: 10.2224/sbp.2016.44.6.923

[44] Schaufeli WB, Bakker AB, Salanova M. The Measurement of Work Engagement With a Short Questionnaire. Educational and Psychological Measurement. 2006;66(4):701–16. doi: 10.1177/0013164405282471

[45] Schaufeli WB, Desart S, De Witte H. Burnout Assessment Tool (BAT)-Development, Validity, and Reliability. Int J Environ Res Public Health. 2020;17(24):9495. doi: 10.3390/ijerph17249495 33352940 PMC7766078

[46] Muthén B, Toit SHCD, Spicic D. Robust inference using weighted least squares and quadratic estimating equations in latent variable modeling with categorical and continuous outcomes. 1997. https://www.statmodel.com/download/Article_075.pdf

[47] Brauer K, Ranger J, Ziegler M. Confirmatory Factor Analyses in Psychological Test Adaptation and Development. Psychological Test Adaptation and Development. 2023;4(1):4–12. doi: 10.1027/2698-1866/a000034

[48] Hu L, Bentler PM. Cutoff criteria for fit indexes in covariance structure analysis: Conventional criteria versus new alternatives. Structural Equation Modeling: A Multidisciplinary Journal. 1999;6(1):1–55. doi: 10.1080/10705519909540118

[49] Kline RB. Principles and practice of structural equation modelling. Fourth Edition ed. The Guilford Press. 2016.

[50] Kenny DA. Measuring model fit. 2024. https://davidakenny.net/cm/fit.htm

[51] Gallucci M. Seemingly odd results in mediation analysis. jAMM: jamovi Advanced Mediation Models. 2024.

[52] MacKinnon DP, Krull JL, Lockwood CM. Equivalence of the mediation, confounding and suppression effect. Prev Sci. 2000;1(4):173–81. doi: 10.1023/a:1026595011371 11523746 PMC2819361

[53] Ryan R, Deci E, Vansteenkiste M. Autonomy and autonomy disturbances in self-development and psycho-pathology: Research on motivation, attachment, and clinical process. In: Cicchetti D. Developmental psychopathology, theory and method. Hoboken, NJ: Wiley. 2016. 385–438.

[54] Vansteenkiste M, Ryan RM, Soenens B. Basic psychological need theory: Advancements, critical themes, and future directions. Motiv Emot. 2020;44(1):1–31. doi: 10.1007/s11031-019-09818-1

[55] Vanhee G, Lemmens GMD, Stas L, Loeys T, Verhofstadt LL. Why are couples fighting? A need frustration perspective on relationship conflict and dissatisfaction. Journal of Family Therapy. 2018;40(S1). doi: 10.1111/1467-6427.12126

[56] Deci EL, Ryan RM. The “What” and “Why” of Goal Pursuits: Human Needs and the Self-Determination of Behavior. Psychological Inquiry. 2000;11(4):227–68. doi: 10.1207/s15327965pli1104_01

[57] Ryan RM, Sheldon KM, Kasser T, Deci EL. All goals are not created equal: An organismic perspective on the nature of goals and their regulation. In: Gollwitzer PM, Bargh JA,. Guilford Press. 1996. 7–26.

[58] Crocker J, Park LE. The costly pursuit of self-esteem. Psychol Bull. 2004;130(3):392–414. doi: 10.1037/0033-2909.130.3.392 15122925

[59] Vansteenkiste M, Neyrinck B, Niemiec CP, Soenens B, De Witte H, Van den Broeck A. On the relations among work value orientations, psychological need satisfaction and job outcomes: A self‐determination theory approach. J Occupat & Organ Psyc. 2007;80(2):251–77. doi: 10.1348/096317906x111024

[60] Martela F, Pessi AB. Significant Work Is About Self-Realization and Broader Purpose: Defining the Key Dimensions of Meaningful Work. Front Psychol. 2018;9:363. doi: 10.3389/fpsyg.2018.00363 29632502 PMC5879150

[61] den Broeck AV, Rivkin W, Slemp GR. Motivating employees using self-determination theory. In: Peeters MCW, de Jonge J, Taris T. An introduction to contemporary work psychology. 2 ed. Wiley. 2024.

[62] HCC. Verzuim door stressklachten in vijf jaar met 30% gestegen. Human Capital Care. 2024. https://www.humancapitalcare.nl/artikelen/verzuim-door-stressklachten-in-vijf-jaar-met-30-gestegen

[63] TNO. Factsheet week van de werkstress 2023. Nationale Enquête Arbeidsomstandigheden (NEA). 2023. https://www.oval.nl/cms/public/files/2023-11/tno-factsheet-week-van-de-werkstress-2023.pdf?0c2e780187

[64] Podsakoff PM, MacKenzie SB, Lee J-Y, Podsakoff NP. Common method biases in behavioral research: a critical review of the literature and recommended remedies. J Appl Psychol. 2003;88(5):879–903. doi: 10.1037/0021-9010.88.5.879 14516251

[65] Podsakoff PM, MacKenzie SB, Podsakoff NP. Sources of method bias in social science research and recommendations on how to control it. Annu Rev Psychol. 2012;63:539–69. doi: 10.1146/annurev-psych-120710-100452 21838546

[66] van Oostrom S, Soeter M, van der Noordt M, van Zon S, van Moll E, Hengel KO. De impact van maatschappelijke ontwikkelingen op de ‘psychosociale arbeidsbelasting’ van werkenden. 2024. https://www.rivm.nl/publicaties/impact-van-maatschappelijke-ontwikkelingen-op-psychosociale-arbeidsbelasting-van-werkenden

[67] Bloom N, Han R, Liang J. Hybrid working from home improves retention without damaging performance. Nature. 2024;630(8018):920–5. doi: 10.1038/s41586-024-07500-2 38867040 PMC11208135

[68] Hopkins J. Managing the Right to Disconnect—A Scoping Review. Sustainability. 2024;16(12):4970. doi: 10.3390/su16124970

[69] Oakman J, Kinsman N, Stuckey R, Graham M, Weale V. A rapid review of mental and physical health effects of working at home: how do we optimise health?. BMC Public Health. 2020;20(1):1825. doi: 10.1186/s12889-020-09875-z 33256652 PMC7703513

